# Supplementary material for: Machine Learning Accelerates Crystallization for Structure Determination
Source: Angew Chem Int Ed Engl. 2026 May 4;65(25):e1218503. doi: 10.1002/anie.1218503 (PMC13266958; doi:10.1002/anie.1218503)

checkCIF/PLATON report

Structure factors have been supplied for datablock(s) lcz056

THIS REPORT IS FOR GUIDANCE ONLY. IF USED AS PART OF A REVIEW PROCEDURE FOR PUBLICATION, IT SHOULD NOT REPLACE THE EXPERTISE OF AN EXPERIENCED CRYSTALLOGRAPHIC REFEREE.

No syntax errors found. CIF dictionary Interpreting this report

Datablock: lcz056

Bond precision: C-C = 0.0088 A Wavelength=1.54184

Cell:	a=12.2255(1)	b=16.8622(2)	c=22.5277(2)
	alpha=90	beta=90	gamma=90
Temperature:	100 K		

	Calculated	Reported
Volume	4644.06(8)	4644.06(8)
Space group	P 21 21 21	P 21 21 21
Hall group	P 2ac 2ab	P 2ac 2ab
Moiety formula	C41 H23 Ag3 F18 N6 O8	C15 H3 Ag3 F18 N6, 2(C13 H10 O4)
Sum formula	C41 H23 Ag3 F18 N6 O8	C41 H23 Ag3 F18 N6 O8
Mr	1393.27	1393.26
Dx,g cm-3	1.993	1.993
Z	4	4
Mu (mm-1)	11.260	11.260
F000	2712.0	2712.0
F000’	2724.37	
h,k,lmax	15,21,28	15,21,28
Nref	9957[5509]	9225
Tmin,Tmax	0.206,0.231	0.880,1.000
Tmin’	0.132	

Correction method= # Reported T Limits: Tmin=0.880 Tmax=1.000
AbsCorr = MULTI-SCAN

```
Data completeness= 1.67/0.93       Theta(max)= 77.979

                                             wR2(reflections)=
R(reflections)= 0.0285( 8885)                0.0735( 9225)

S = 1.100                Npar= 689
```

The following ALERTS were generated. Each ALERT has the format
test-name_ALERT_alert-type_alert-level.
Click on the hyperlinks for more details of the test.

Alert level C

```
PLAT042_ALERT_1_C Calc. and Reported MoietyFormula Strings  Differ     Please Check
              Calc: C41 H23 Ag3 F18 N6 O8
              Rep.: C15 H3 Ag3 F18 N6, 2(C13 H10 O4)
PLAT090_ALERT_3_C Poor Data / Parameter Ratio (Zmax > 18) ........       7.72 Note
PLAT213_ALERT_2_C Atom F00F            has ADP max/min Ratio .....        3.4 prolat
PLAT213_ALERT_2_C Atom F00H            has ADP max/min Ratio .....        3.1 prolat
PLAT213_ALERT_2_C Atom F014            has ADP max/min Ratio .....        3.4 prolat
PLAT213_ALERT_2_C Atom F01X            has ADP max/min Ratio .....        3.7 prolat
PLAT220_ALERT_2_C NonSolvent   Resd 1  F   Ueq(max)/Ueq(min) Range        3.2 Ratio
PLAT342_ALERT_3_C Low Bond Precision on  C-C Bonds ..............    0.00875 Ang.
PLAT911_ALERT_3_C Missing FCF Refl Between Thmin & STh/L=    0.600          5 Report
               12  0  0,   0 20  0,   0 20  1,   0  0  2,   9  0 21,
```

Alert level G

```
PLAT143_ALERT_4_G s.u. on c - Axis Small or Missing .............    0.00020 Ang.
PLAT242_ALERT_2_G Low    'MainMol' Ueq as Compared to Neighbors of       C01H Check
PLAT242_ALERT_2_G Low    'MainMol' Ueq as Compared to Neighbors of       C01K Check
PLAT242_ALERT_2_G Low    'MainMol' Ueq as Compared to Neighbors of       C01Z Check
PLAT242_ALERT_2_G Low    'MainMol' Ueq as Compared to Neighbors of       C020 Check
PLAT242_ALERT_2_G Low    'MainMol' Ueq as Compared to Neighbors of       C022 Check
PLAT242_ALERT_2_G Low    'MainMol' Ueq as Compared to Neighbors of       C023 Check
PLAT398_ALERT_2_G Deviating  C-O-C   Angle From 120 for O00K     .      105.0 Degree
PLAT398_ALERT_2_G Deviating  C-O-C   Angle From 120 for O00P     .      105.3 Degree
PLAT434_ALERT_2_G Short Inter HL..HL Contact F00H     ..F00J     .       2.79 Ang.
                                              1+x,y,z  =      1_655 Check
PLAT434_ALERT_2_G Short Inter HL..HL Contact F00H     ..F00O     .       2.82 Ang.
                                      1/2+x,3/2-y,1-z  =      4_566 Check
PLAT720_ALERT_4_G Number of Unusual/Non-Standard Labels .........         99 Note
              Ag01    Ag02    Ag03    O004    O005    O006    F007    F008
              O009    O00A    F00B    F00C    F00D    O00E    F00F    N00G
              F00H    F00I    F00J    O00K    N00L    N00M    F00N    F00O
              O00P    N00Q    F00R    F00S    N00T    F00U    F00V    C00W
              C00X    H00X    N00Y    C00Z    C010    C011    H01A    H01B
              H01C    C012    C013    F014    C015    H015    C016    H016
              C017    F018    C019    H019    C01A    H01D    C01B    C01C
              H01E    H01F    H01H    C01D    C01E    H01I    H01J    H01K
              C01F    C01G    H01G    C01H    C01I    C01J    C01K    C01L
              C01M    C01N    C01O    C01P    C01Q    C01R    C01S    H01S
              C01T    H01T    C01U    C01V    H01V    C01W    H01W    F01X
              C01Y    C01Z    C020    C021    H021    C022    C023    C024
              H02A    H02B    H02C
```

```
PLAT883_ALERT_1_G Absent Datum for _atom_sites_solution_primary ..     Please Do !
PLAT912_ALERT_4_G Missing # of FCF Reflections Above STh/L=  0.600        185 Note
PLAT941_ALERT_3_G Average HKL Measurement Multiplicity ..........         4.3 Low
PLAT969_ALERT_5_G The 'Henn et al.' R-Factor-gap value ..........       1.710 Note
              Predicted wR2: Based on SigI**2  4.30 or SHELX Weight  6.68
PLAT978_ALERT_2_G Number C-C Bonds with Positive Residual Density.          0 Info
```

---

   0 **ALERT level A** = Most likely a serious problem - resolve or explain
   0 **ALERT level B** = A potentially serious problem, consider carefully
   9 **ALERT level C** = Check. Ensure it is not caused by an omission or oversight
  17 **ALERT level G** = General information/check it is not something unexpected

```
   2 ALERT type 1 CIF construction/syntax error, inconsistent or missing data
  16 ALERT type 2 Indicator that the structure model may be wrong or deficient
   4 ALERT type 3 Indicator that the structure quality may be low
   3 ALERT type 4 Improvement, methodology, query or suggestion
   1 ALERT type 5 Informative message, check
```

Validation response form

Please find below a validation response form (VRF) that can be filled in and pasted into your CIF.

```
# start Validation Reply Form
_vrf_PLAT042_lcz056
;
PROBLEM: Calc. and Reported MoietyFormula Strings  Differ     Please Check
RESPONSE: ...
;
_vrf_PLAT090_lcz056
;
PROBLEM: Poor Data / Parameter Ratio (Zmax > 18) ........       7.72 Note
RESPONSE: ...
;
_vrf_PLAT213_lcz056
;
PROBLEM: Atom F00F            has ADP max/min Ratio .....        3.4 prolat
RESPONSE: ...
;
_vrf_PLAT220_lcz056
;
PROBLEM: NonSolvent   Resd 1  F   Ueq(max)/Ueq(min) Range        3.2 Ratio
RESPONSE: ...
;
_vrf_PLAT342_lcz056
;
PROBLEM: Low Bond Precision on  C-C Bonds ..............    0.00875 Ang.
RESPONSE: ...
;
_vrf_PLAT911_lcz056
;
PROBLEM: Missing FCF Refl Between Thmin & STh/L=    0.600          5 Report
RESPONSE: ...
;
# end Validation Reply Form
```

It is advisable to attempt to resolve as many as possible of the alerts in all categories. Often the minor alerts point to easily fixed oversights, errors and omissions in your CIF or refinement strategy, so attention to these fine details can be worthwhile. It is up to the individual to critically assess their own results and, if necessary, seek expert advice.

PLATON version of 17/09/2025; check.def file version of 17/09/2025

---

duplicate check

No duplication found

Datablock lcz056 - ellipsoid plot

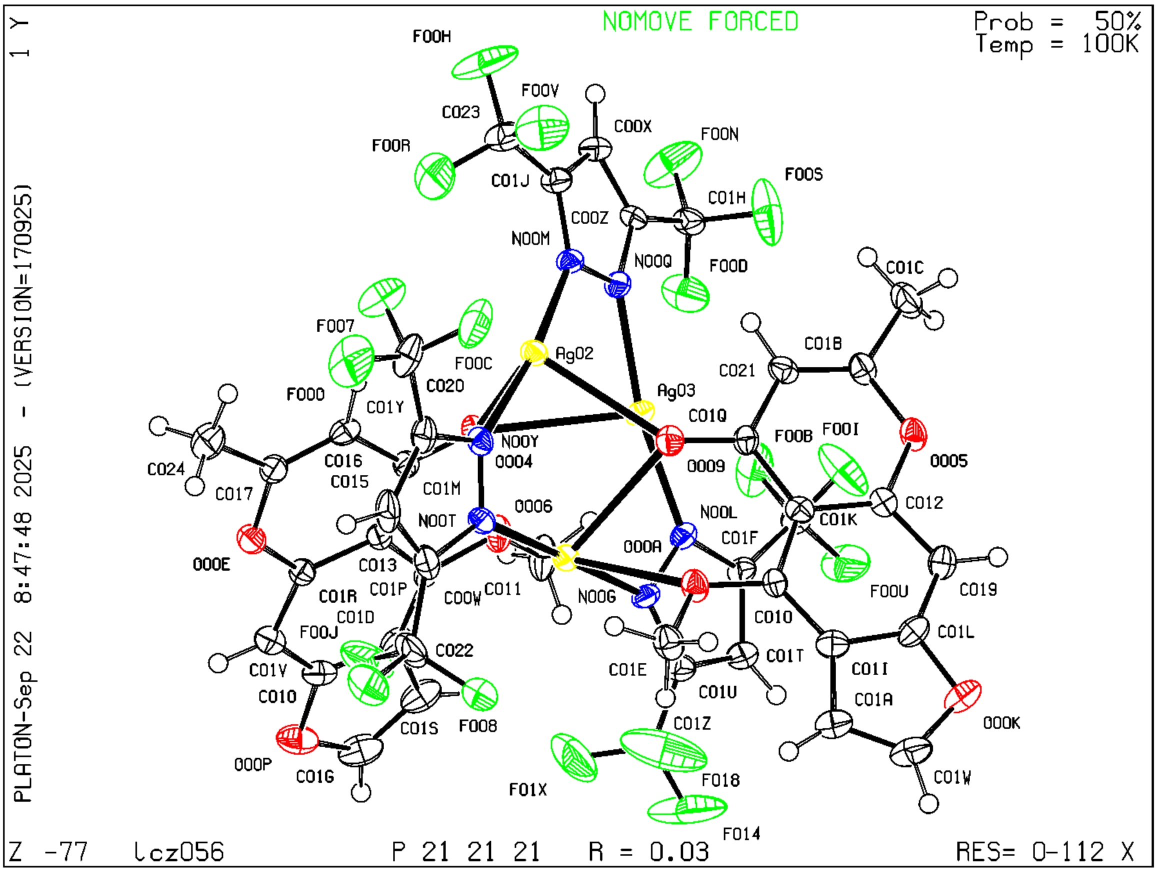

Supplement: Supplementary file 2 — Supporting File 2: anie72462‐sup‐0002‐DataFile.zip. [file ANIE-65-e1218503-s002.zip › supplementary_materials_crystallographic_data submit.zip!/cif_and_checkcif(1-114)/Ag3Pz3_56/Ag3Pz3_56_checkcif.pdf]
